# Apatinib treatment for KIT- and KDR-amplified angiosarcoma: a case report

**DOI:** 10.1186/s12885-018-4523-2

**Published:** 2018-05-31

**Authors:** Lishu Yang, Lizhu Liu, Bo Han, Wei Han, Meng Zhao

**Affiliations:** 1 Department of Oncology, First Affiliated Hospital of Harbin Medical University, Harbin, 150001 China; 2 Department of Pathology, First Affiliated Hospital of Harbin Medical University, Harbin, 150001 China; 3 3D Medicine Inc. Shanghai, Shanghai, 201114, People’s Republic of China

**Keywords:** Apatinib, Angiosarcoma, KDR, Gene-amplified

## Abstract

**Background:**

Metastatic or relapsed angiosarcoma has a poor prognosis and the efficacy of conventional chemotherapy is often limited. Apatinib, a novel tyrosine kinase inhibitor (TKI) targeting vascular endothelial growth factor receptor-2 (VEGFR2), has been approved for the treatment of advanced gastric cancer.

**Case presentation:**

Herein, we report a patient with advanced angiosarcoma, who received apatinib at a daily dose of 250 to 725 mg, resulting in a partial response for three months, which may be related to Kinase Insert Domain Receptor (KDR) gene amplification.

**Conclusion:**

Our experience reported here indicated that apatinib may be a useful therapeutic option for treatment of patients with advanced angiosarcoma.

## Background

Patients with metastatic or relapsed angiosarcoma initially respond well to cytotoxic chemotherapy, however improvements are often short-lived, with a median time to progression averaging four to five months [1, 2]. Therefore, there is an urgent need for developing efficacious and safe therapies for the treatment of angiosarcoma. Vascular endothelial growth factor receptors (VEGFRs)–targeted therapy has been suggested as a promising approach to the effective treatment of angiosarcoma [3]. Unfortunately, clinical trials with bevacizumab or sorafenib monotherapies in patients with angiosarcoma have thus far shown limited efficacy, with response rates reported as 9 to 14% [4, 5]. Herein, we report a patient with a scalp angiosarcoma who responded to apatinib, a novel VEGFR2 inhibitor. We suggest that apatinib should be considered in the management of advanced angiosarcoma.

## Case presentation

**Fig. 1:**
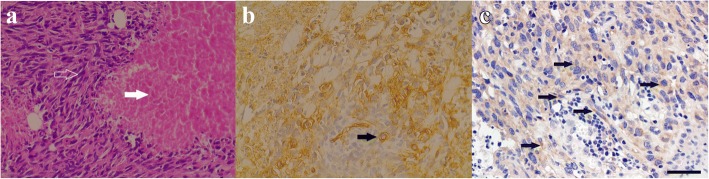
Hematoxylin and eosin (H&E) staining of a tumor section. **a** × 400; The tumor cells are spindle shaped with obvious heteromorphism(white arrow), and large areas of necrosis(hollow arrow) are visible; **b** × 400. CD34 cell membrane positive(black arrow). **c** × 400. Immunohistochemistry demonstrates expression of VEGFR2 with color brown (black arrow)

A 67-year-old Chinese man appeared with a growing nodular, vascular tumor (enlarging from 0.5 × 0.5 cm to 4 × 3 cm in nine months) with pruritus and ulcers in the left parietal region of the scalp. The patient presented to the neurosurgery clinic of our hospital in November 2015. A computed tomography (CT) scan of the chest, abdomen, and pelvis showed no evidence of metastatic disease. The patient underwent macroscopic radical resection of the tumor and free skin graft repair. Histopathology analysis revealed diffuse distribution of tumor cells with an invasive growth pattern in the epidermis and dermis. Tumor cells were oval and spindle-shaped and showed obvious cellular atypia. Lumen-like structures and massive tumor necrosis was observed (Fig. 1) as angiosarcoma of corium and subcutaneous with infiltration into the muscles and the soft tissues. Histoimmunnochemistry analysis revealed a typical vascular endothelium CD31 (+) and CD34 (+), and lymphatic endothelium D2–40 (+) with a high Ki-67 index (50%). A computed tomography (CT) scan of the chest, abdomen, and pelvis showed no evidence of metastatic disease. The patient refused to undergo adjuvant chemotherapy and/or radiotherapy as recommended. One month after surgery, ^18^F-FDG PET/CT scan showed evidence of metastasis to liver and bone, with local recurrence occurred two months later. The patient was hospitalized in May 2016 due to scalp angiosarcoma recurrence at six months post-surgery. The patient did not experience any systemic symptoms during the post-operative period. With respect to the patient’s medical history, the patient had a history of hypertension for five years, but denied any family history of malignancy. Physical examination showed pink nodules with ulceration in the parietal and frontal regions of the scalp, and nodular lesions anterior to the left ear. A CT scan of chest and abdomen showed multiple solid nodules in the lungs and liver. Radiotherapy was not considered necessary due to widespread recurrent and metastatic diseases. This patient refused chemotherapy.

**Fig. 2:**
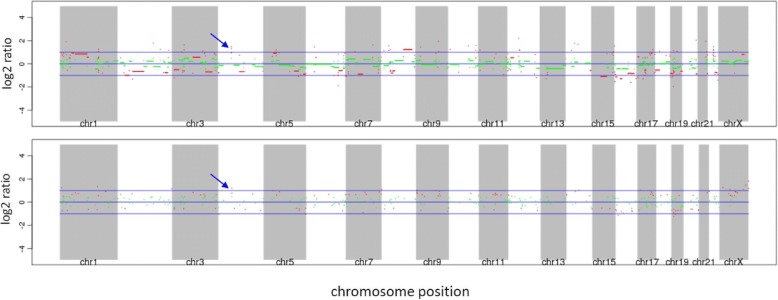
Genomic profiling identifies amplification of KDR (VEGFR2) and KIT4. Genomic profiling by a hybrid capture-based next-generation sequencing assay was performed on the resected specimen tissue to a coverage depth of 561×, revealing 4 distinct genomic alterations, including *KIT* amplification (8 copies) and *KDR* amplification (8 copies)(blue arrow, *KDR* and *KIT* are relatively close to the genome, so they can’t be separated.). Copy number was determined by modeling copy variation and aneuploidy across the genome, and was compared with process-matched normal controls

**Fig. 3:**
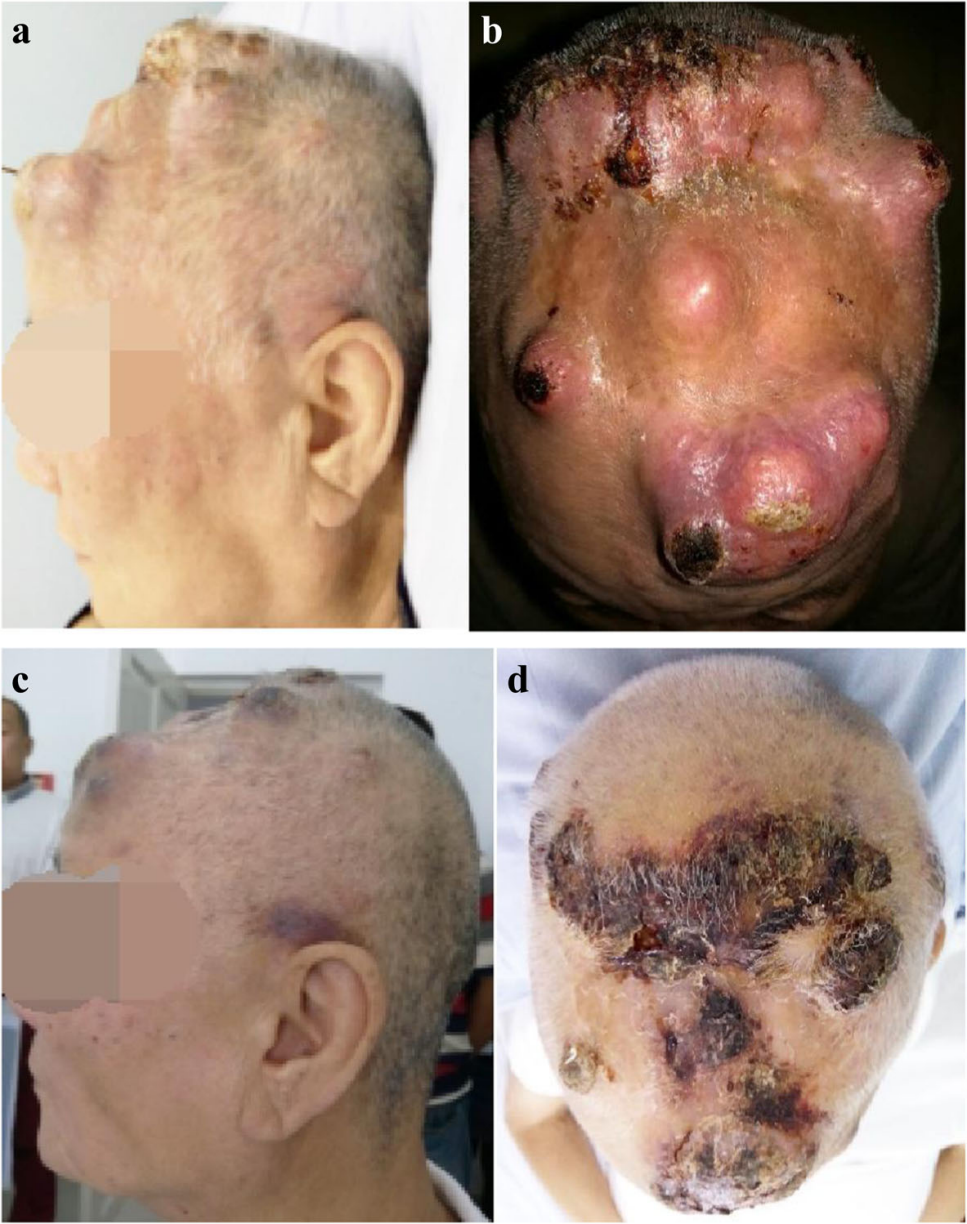
Extensive cutaneous angiosarcoma of the scalp. (**a** and **b**) Before initiation of apatinib. **c** After three weeks of treatment with oral apatinib at 675 mg daily (**d**) After eight weeks of treatment with oral apatinib at 675 mg daily

**Fig. 4:**
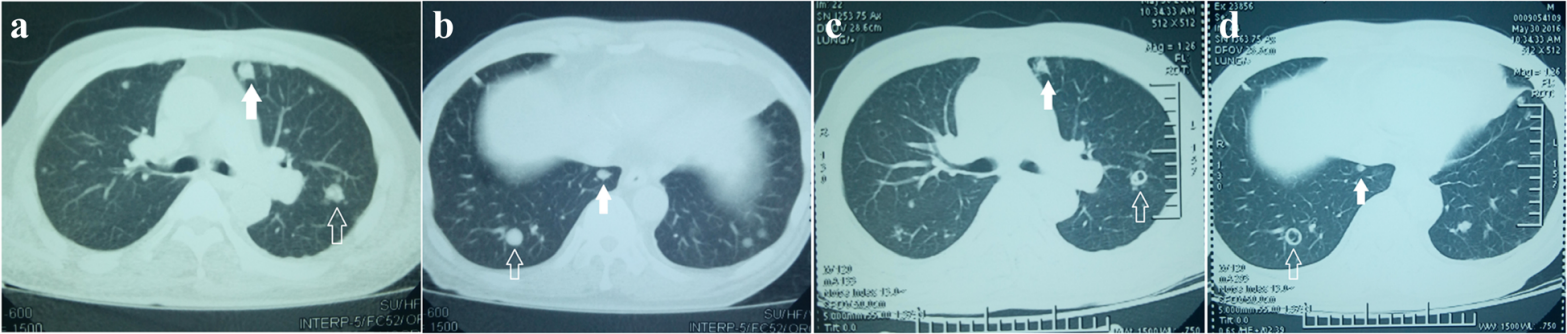
CT scans showing significant reduction of solid nodules(white arrows) and transform from solid nodules before treatment(hollow arrow) (**a** and **b**) to cystic nodules(hollow arrow) after 3 weeks of treatment with apatinib (**c** and **d**)

Considering that KDR gene amplification [2], KDR gene mutation and strong KDR (VEGFR2) expression in angiosarcoma [6, 7] have been previously reported, apatinib was given after the patient provided written informed consent. Apatinib was administrated at a dose of 250 mg/day in May 2016, increasing to 675 mg/day after five days. Resected angiosarcoma tissue from the scalp was analyzed for gene mutations, and demonstrated *KIT* amplification (8 copies), *KDR* amplification (8 copies) (Fig. 2), *RB1* codon 251 arginine mutated to a stop codon, and *TP53* codon 195 isoleucine mutated to threonine with a reduced copy number. After three weeks of apatinib treatment, the swelling of scalp and forehead was markedly reduced (Fig. 3) and the CT scan of chest showed significant reduction of solid nodules and transformation of solid nodules into cystic nodules (Fig. 4). Physical examination conducted in July 2016 showed no progressive disease, a closed pneumothorax of the right thoracic cavity was found after 10 weeks of apatinib administration, with suspected side-effects, such as glossodynia, odynophagia, upper abdominal pain, melena, hemoptysis, and delayed healing of the lesion of scalp after tumor regression. Medication was stopped for one week in order to promote the recovery of the left-sided pneumothorax after 11 weeks of administration of apatinib. The patient then received a reduced daily dose of 425 mg apatinib for three weeks. When closure of the pneumothorax of right thoracic happened, the medication had to be interrupted again, and disease progression was observed in scalp, and lungs and liver proven by CT scan. Further therapy was limited because of his severe comorbidities. The patient subsequently died after his partial response maintained for three months.

## Discussion

The treatment with apatinib in our patient resulted in a three-month partial remission (PR). The duration of the PR achieved should be considered acceptable. Based on previous reports, the median progression-free survival time (PFS) and response rate in angiosarcomas are with 3.8 months and 14% for sorafenib [8] and three months and 9% for bevacizumab, respectively [9].

Angiosarcoma is a malignant neoplasm of vascular-origin derived from endothelial cells [7]. The NCCN Clinical Practice Guidelines in Oncology (NCCN Guidelines) for Soft Tissue Sarcoma recommends surgery and radiotherapy as the primary mode of treatment for local control of angiosarcoma. Systemic therapy is essential due to high local recurrence rates and distant metastasis risk. Patients with metastatic or relapsed disease often respond well to cytotoxic chemotherapy initially, however the median time to progression is short, often four to five months [10, 11].

The use of cytotoxic agents and targeted therapies forms the mainstay of therapy for patients with metastatic or unresectable disease [12] As a novel targeted therapeutic agent, apatinib targets VEGFR signaling, which is frequently implicated in angiosarcoma. Indeed, VEGFR2 regulates angiogenesis [13] and inappropriate activation of VEGFR signaling is critical in the development and progression of angiosarcoma [2, 6, 7, 14]. Activating base substitutions in VEGFR2 [7] and VEGFR1–3 overexpression have been demonstrated in patients with angiosarcoma [13, 15]. In order to explore the relationship between KDR gene amplification and VEGFR2 expression, we carried out immunohistochemical detection of VEGFR-2 in the resected tumor tissues, and found a moderate degree of expression (Fig. 1c).

Apatinib is a small-molecule TKI that highly selectively binds to and strongly inhibits VEGFR-2, decreasing VEGF-mediated endothelial cell migration, proliferation, and tumor microvascular formation. It has been shown to be a novel therapeutic option in a variety of tumor types [16]. In a Phase I study, dose escalation of apatinib was conducted from 250 to 1000 mg in patients with solid tumors aged between 18 and 70 years, with the study demonstrating a maximum tolerated dose of 850 mg once daily. The most frequently observed drug-related adverse events were hypertension (69.5%), proteinuria (47.8%), and hand-foot syndrome (45.6%) [1].

Identifying patients with an increased likelihood of response to therapy is a major challenge in the clinic. Identification of genomic alterations may help further sub-classify tumors. Based on our case, there was a possible correlation between responsiveness to apatinib and amplification of *KDR*. Pre-clinical studies have shown that KDR activation variants are more sensitive to sunitinib and sorafenib [7]; indeed, one of the targets of apatinib is KDR [17]. In addition to our findings, there have been some previously published reports with similar findings. Colorectal cancer patients with pulmonary metastases who had a VEGFR2 mutation responded to apatinib treatment [1]. Response to sunitinib has been reported in patients with KIT mutation [3] and in patients with KDR mutations [18]. The IC_50_ for apatinib with respect to VEGFR2 is 0.001 M, whereas the IC_50_ for sunitinib is 0.005 M [19] suggesting that apatinib is highly selective. Our case may represent a subset of highly apatinib-responsive angiosarcomas characterized by *KDR* amplification.

Our patient presented some common adverse events, such as hypertension, proteinuria, and hand-foot syndrome [1], which were not severe and therefore did not require dose reduction or withdrawal of the therapy. However, we also observed other side effects, including glossodynia, odynophagia, upper abdominal pain, melena, hemotpyssis, delayed healing of scalp lesions, and a pneumothorax. These symptoms seriously reduced oral intake and thus worsened the patient’s physical condition. The pneumothorax might be the result of tumor regression and indeed, eventually led to withdrawal of the medication. It may also be a feasible choice to maintain a low dose rather than a standard dose if the dose of 250 mg can achieve a significant tumor regression. When drug resistance occurs, the patient might still have an opportunity to receive other targeted drug treatment such as Pazopanib [2], Sunitinib [3], sorafenib [8], and bevacizumab [9]. Therefore, individualized dose scheduling should be considered in order to achieve a balance between tumor regression and tissue repair to expect a longer period of remission.

## Conclusion

The present case demonstrated the potential therapeutic effects of apatinib in patients with metastatic angiosarcoma. Future clinical studies are required to identify a subset of patients with angiosarcomas with well-defined genomic variants sensitive to apatinib. Prospective clinical trials accompanied by comprehensive genomic profiling are needed. Additionally, pharmacogenomics studies will be needed to help define individual dosing and scheduling at the beginning of the therapy and aid in dose adjustment based on patient responses and safety profiles.

## Abbreviations

KDR: Kinase Insert Domain Receptor
TKI: Tyrosine kinase inhibitor
